# Incidence of hip fracture in Saudi Arabia and the development of a FRAX model

**DOI:** 10.1007/s11657-022-01085-x

**Published:** 2022-04-02

**Authors:** Yousef A. L. Saleh, Riad A. Sulimani, Shaker Alomary, Yassmeen I. Alnajjar, Liesbeth Vandenput, Enwu Liu, Mattias Lorentzon, Nicholas C. Harvey, Eugene V. McCloskey, Helena Johansson, John A. Kanis, the Saudi FRAX Study Group, Nasser M. Al-Daghri, Abdelgadier Ibrahim Jamo, Abdullah Ahmed Hawsawi, Fatima Ali Mohamed, Talha Mohammedsaeed Khojah, Eman Abdulrahman Sheshah, Waleed A. Hashem, Abdulgani Omar Hijazi, Samer Merei Kanani, Ashwag Saleh Alfagih, Kamil Muslim Albouri, Osama Fawaz Alsobyhy, Mohammed Zayed Almutairi, Mussa Hussain Almalki, Nadia Abd Elhamid Kassem, Mutaz Mohamed Ali, Fahad A. Alamri, Fahad Mohammed Alshahrani, Hanan Mohammed AlRayes, Mir Sadat-Ali, Mohammed Abdulrahman Alharbi, Mohammed AlShaker, Mona A. Fouda, Salwa Berlian Alaidarous, Mohammed Almohaya, Najla Alfateh Saleh, Soad Saleh

**Affiliations:** 1 College of Medicine, King Saud Bin Abdulaziz University for Health Sciences, Riyadh, Saudi Arabia; 2 King Abdullah International Medical Research Center, Riyadh, Saudi Arabia; 3 Department of Medicine, King Abdulaziz Medical City, Riyadh, Saudi Arabia; 4 Ministry of National Guard-Health Affairs, Riyadh, Saudi Arabia; 5 Biomarkers of Chronic Diseases, Biochemistry Department, College of Science, King Saud University, Riyadh, Saudi Arabia; 6 Department of Medicine, College of Medicine, King Khaled University Hospital, King Saud University Medical City, King Saud University, Riyadh, Saudi Arabia; 7 Health Programs and Chronic Diseases, Ministry of Health, Riyadh, Saudi Arabia; 8 Health Programs and Chronic Diseases, Osteoporosis Program, Ministry of Health, Riyadh, Saudi Arabia; 9 Mary McKillop Institute for Health Research, Australian Catholic University, Melbourne, Australia; 10 Department of Internal Medicine and Clinical Nutrition, Institute of Medicine, Sahlgrenska Osteoporosis Centre, Sahlgrenska Academy, University of Gothenburg, Gothenburg, Sweden; 11 Sahlgrenska Osteoporosis Centre, Institute of Medicine, University of Gothenburg, Gothenburg, Sweden; 12 Geriatric Medicine, Region Västra Götaland, Sahlgrenska University Hospital, Mölndal, Sweden; 13 MRC Lifecourse Epidemiology Centre, University of Southampton, Southampton, UK; 14 NIHR Southampton Biomedical Research Centre, University of Southampton and University Hospital Southampton NHS Foundation Trust, Southampton, UK; 15 Centre for Metabolic Bone Diseases, University of Sheffield, Sheffield, UK; 16 Department of Oncology and Metabolism, Mellanby Centre for Musculoskeletal Research, University of Sheffield, Sheffield, UK

**Keywords:** FRAX, Fracture probability, Osteoporosis epidemiology, Hip fracture, Saudi Arabia

## Abstract

***Summary*:**

A prospective hospital-based survey in representative regions of Saudi Arabia determined the incidence of fractures at the hip. The hip fracture rates were used to create a FRAX® model to facilitate fracture risk assessment in Saudi Arabia.

**Objective:**

This paper describes the incidence of hip fracture in the Kingdom of Saudi Arabia that was used to characterize the current and future burden of hip fracture, to develop a country-specific FRAX® tool for fracture prediction and to compare fracture probabilities with neighbouring countries.

**Methods:**

During a 2-year (2017/2018) prospective survey in 15 hospitals with a defined catchment population, hip fractures in Saudi citizens were prospectively identified from hospital registers. The number of hip fractures and future burden was determined from national demography. Age- and sex-specific incidence of hip fracture and national mortality rates were incorporated into a FRAX model for Saudi Arabia. Fracture probabilities were compared with those from Kuwait and Abu Dhabi.

**Results:**

The incidence of hip fracture applied nationally suggested that the estimated number of hip fractures nationwide in persons over the age of 50 years for 2015 was 2,949 and is predicted to increase nearly sevenfold to 20,328 in 2050. Hip fracture rates were comparable with estimates from Abu Dhabi and Kuwait. By contrast, probabilities of a major osteoporotic fracture or hip fracture from the age of 70 years were much lower than those seen in Abu Dhabi and Kuwait due to higher mortality estimates for Saudi Arabia.

**Conclusion:**

A country-specific FRAX tool for fracture prediction has been developed for Saudi Arabia which is expected to help guide decisions about treatment.

## Introduction

Osteoporosis is a common, chronic, and costly condition; its principal clinical consequence is fracture. In Europe, the annual cost of fractures associated with osteoporosis exceeded € 37 billion in 2019 [1]; disability due to osteoporosis was greater than that caused by any single cancer, with the exception of lung cancer, and was comparable or greater than that lost to a variety of chronic noncommunicable diseases, such as rheumatoid arthritis, asthma, and high blood pressure-related heart disease [2–4]. Fortunately, a wide variety of treatments is available that favourably affect bone mass and thereby decrease the risk of fractures associated with osteoporosis [5]. The use of such interventions by health care practitioners is assisted by instruments that assess patients’ fracture risk to optimise clinical decisions about prevention and treatment. The most widely used web-based tool FRAX® (https://www.sheffield.ac.uk/FRAX/) meets these requirements and computes the 10-year probability of fragility fractures based on several common clinical risk factors and, optionally a DXA scan result [6, 7]. FRAX models are available for 78 countries in 2021 covering more than 80% of the world population at risk [8] and have been incorporated into more than 100 guidelines worldwide [9].

The availability of FRAX has stimulated studies that can be used for the generation of new FRAX models. Specific examples include Brazil, Mexico, and Turkey [9]. Currently, the Gulf Cooperation Council (GCC) countries’ osteoporosis societies recommend the use of Kuwait or Abu Dhabi versions for GCC states that do not have a FRAX model, Saudi Arabia included [10]. Hence, the broad aim of the present study was to provide epidemiological information on fracture risk so that a FRAX model could be created for Saudi Arabia. The present report describes the incidence of hip fracture in the Kingdom of Saudi Arabia, the current and future burden of hip fracture, the development of a country-specific FRAX® tool for fracture prediction and a comparison of fracture probabilities with neighbouring countries.

The country-specific FRAX model is now available online; https://www.sheffield.ac.uk/FRAX/tool.aspx?country=84).

## Methods

The Kingdom of Saudi Arabia constitutes the bulk of the Arabian Peninsula with a surface area of approximately 2,150,000 km^2^ (830,000 square miles). It is bordered by Jordan, Iraq, Kuwait, Qatar, Bahrain, the United Arab Emirates, Oman, and Yemen. Saudi Arabia also has one of the world’s youngest populations, with approximately 41% of its population of 34.2 million being under 25 years old. Conversely, less than 10% of the population has an age of 55 or more years [11].

The study to define the incidence of hip fracture was developed in collaboration between the Ministry of Health represented by the Directorate for Prevention of Osteoporosis and the Saudi Osteoporosis Society. Representative hospitals were selected from various parts of the country to cover all geographic regions (Riyadh, Dammam, Makkah Al Mokaramah, Jazan, Al-Jouf, Al-Baha, Tabouk, Najran, and Hail). A total of 15 hospitals that admitted and operated on patients with hip fractures were selected. The choice of hospital was determined by the willingness of a research officer to collaborate. Champions in these institutions were identified and a meeting was convened in Riyadh to discuss and explain the data collection process. Case report forms were developed to record the patient's age, sex, residence, date, character of injury and ICD-10 code (S72.0, S72.1, S72.2). The group thereafter communicated through social media and met again after 8 months to discuss the progress of data collection, review the accuracy of hip fracture recording, and meet any challenges.

Data on low-energy hip fracture were collected on Saudi citizens age 45 years and above with low trauma fractures during 2017 and 2018. Hip fractures associated with road traffic accidents and other major trauma was excluded. Pathological fractures secondary to malignancy and metabolic bone disorders were also excluded. To avoid double-counting, further admissions for the same fracture site in the observation time were excluded.

It was estimated that the catchment hospitals captured 12.82% of the total Saudi population based on a Ministry of Health assessment of the total number of orthopaedic beds dealing with hip fracture cases. Thus, the catchment population comprised 4 million Saudi nationals (2,031,601 men and 1,952,734 women) age 45 years or older.

The age and sex-specific incidence in 2017/2018 was applied to the population in 2015 to estimate the number of hip fractures nationwide. Additionally, future projections were estimated up to 2050 assuming that the age- and sex-specific incidence remained stable. Population demography was taken from the United Nations using the medium variant for fertility [12].

The data on hip fracture were used to construct the FRAX model. For other major osteoporotic fractures (clinical spine, forearm, and humeral fractures), it was assumed that the age- and sex-specific ratios of these fractures to hip fracture risk found in Sweden were comparable to those in Saudi Arabia. This assumption has been used for many of the FRAX models with incomplete epidemiological information. Available information suggests that the age- and sex-stratified pattern of fracture is very similar in the Western world, Australia, and Eastern Europe [13–16].

The development and validation of FRAX have been extensively described [6, 7]. The risk factors used were based on a systematic set of meta-analyses of population-based cohorts worldwide and validated in independent cohorts with over 1 million patient years of follow-up. The construct of the FRAX model for Saudi Arabia retained the beta coefficients of the risk factors in the original FRAX model with the incidence rates of hip fracture and mortality rates for Saudi Arabia. National mortality rates used data from the Household Health Survey, 2018 [17]. Ten-year fracture probabilities were compared to those of the neighbouring countries where a FRAX model was available (Kuwait and United Arab Emirates (Abu Dhabi)).

In order to compare hip fracture probabilities with those of other regions of the world, the remaining lifetime probability of hip fracture from the age of 50 years was calculated for men and women, as described previously [18]. In the present analysis, values for Saudi Arabia were compared with those of Abu Dhabi, Botswana, China (Hong Kong), Bulgaria, Canada, Denmark, Finland, France, Germany, Greece, Hungary, Iran, Kazakhstan, Kuwait, Moldova, Morocco, Netherlands, Poland, Portugal, Romania, Russia, Singapore (Indian), South Africa (White and Black), Spain, Sweden, Tunisia, Turkey, Ukraine, the United Kingdom, the USA (Caucasian and Black), and Uzbekistan [19, 20].

## Results

Over the period of two years, 684 low-energy hip fractures were identified in men (*n* = 296) and women (*n* = 388) age 45 years or more. Sites of fracture were neck of femur (43%), pertrochanteric (47%), and subtrochanteric (10%).

The crude annual incidence of low-energy hip fracture in individuals age 45 years or more was 77.5/100,000 in women, and 56.8/100,000 in men (female/male ratio = 1.4). Hip fracture incidence increased with the age up to the age of 90 years in both men and women (Table 1). The incidence in women rose more steeply with age than in men, although not statistically significant, and age-specific fracture rates were higher in women than in men from the age of 55 years although statistically significant only for age 55–59 years.

**Table 1:** Population at risk (2017), number of hip fractures (2017/2018) and annual hip fracture incidence (per 100,000) with 95% confidence intervals in the male (M) and female (F) population

Age (years)	Population		Number of hip fractures		*M*		*F*	
	*M*	*F*	*M*	*F*	Incidence	95%CI	Incidence	95%CI
45–49	71,733	67,888	4	3	2.8	0.8–7.1	2.2	0.5–6.5
50–54	57,212	54,091	21	18	18	11–28	17	9.9–26
55–59	44,624	40,987	19	32	21	13–33	39	27–55
60–64	32,327	30,246	24	33	37	24–55	55	38–77
65–69	19,670	20,869	31	48	79	54–112	115	85–153
70–74	14,356	14,739	46	65	160	117–214	221	170–281
75–79	9,358	9,552	58	72	310	235–401	377	295–475
80–84	11,173	11,967	93	117	416	336–510	489	404–586
	260,453	250,339	296	388				

**Fig. 1:**
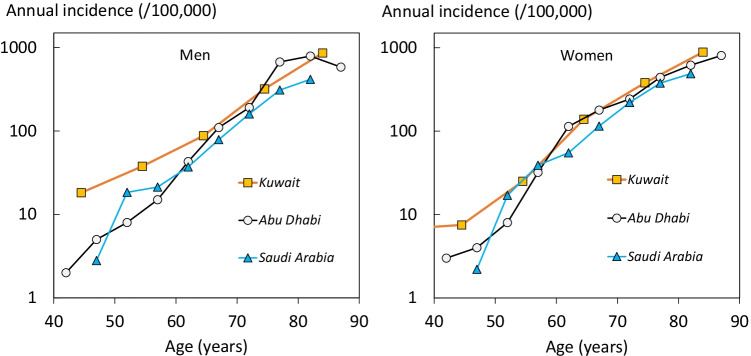
The incidence of hip fracture by age in men (left panel) and women (right panel) in Saudi Arabia, Abu Dhabi, and Kuwait on a logarithmic scale

The incidence of hip fracture in Saudi Arabia was compared with that from Abu Dhabi and Kuwait is shown on a logarithmic scale in Figure 1. Hip fracture rates were rather comparable between these countries.

Assuming that the fracture rates in the areas surveyed were representative for the whole country, the estimated number of hip fractures nationwide in persons over the age of 50 years for 2015 was 2,949 and is predicted to increase nearly 7-fold to 20,328 in 2050 (Table 2).

**Table 2:** Estimated total number of hip fractures in men and in women age 50 years or older in 2015 projected up to 2050 in Saudi Arabia

	2015	2020	2030	2040	2050
Men	1416	1870	3633	6600	10,109
Women	1533	1907	3338	6033	10,219
Men and women	2949	3777	6971	12,633	20,328

**Fig. 2:**
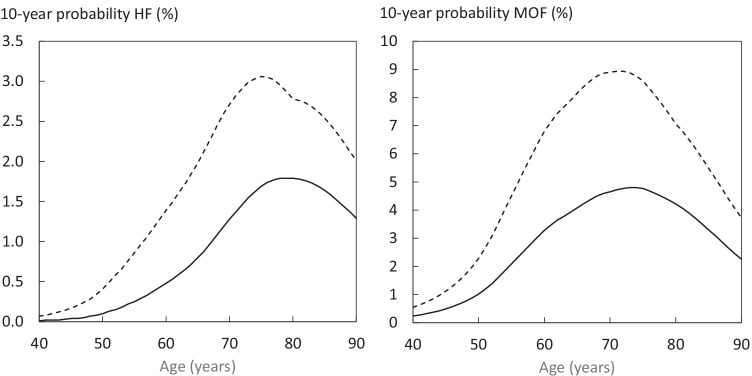
Ten-year probability of a major osteoporotic fracture in women by age. The solid line shows probabilities in women with no clinical risk factors and the dotted line shows probabilities in women with a prior fragility fracture. Body mass index was set at 25 kg/m^2^

Probabilities of a hip fracture and major osteoporotic fracture are shown for women in Figure 2. Probabilities rose with age up to the age of 70 years and decreased thereafter due to the competing effect of mortality.

**Fig. 3:**
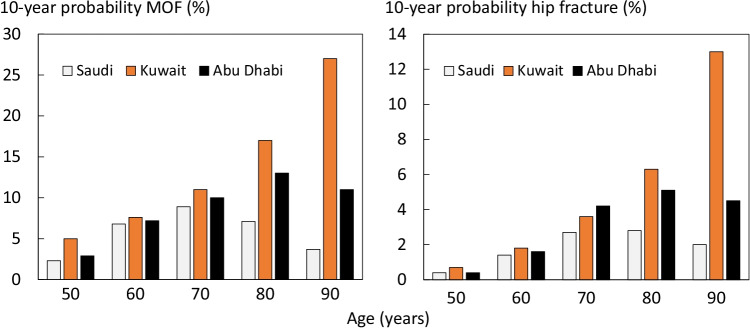
Ten-year probability of a major osteoporotic fracture (left-hand panel) and hip fracture (right) in women with a prior fracture by age from Saudi, Kuwait, and Abu Dhabi. Body mass index set to 25 kg/m^2^

Fracture probabilities in women with a prior fragility fracture are compared with those derived from the FRAX models for Kuwait and Abu Dhabi in Figure 3. At younger ages, fracture probabilities were rather similar between countries but diverged markedly with age. For Kuwait, fracture probabilities increased progressively with age up to the age of 90 years. For Abu Dhabi, fracture probabilities increased up to the age of 80 years. As noted above, probabilities in Saudi women reached a peak at the age of 70 years.

Lifetime probabilities for hip fracture are shown in Table 3. For Saudi Arabia, probabilities were less than 1 in 20, and similar to probabilities in South African blacks and Morocco. As might be expected from the differences in mortality, probabilities were approximately half that estimated for Kuwait and Abu Dhabi.

**Table 3:** Life-time probability of hip fracture in the Qatari population at the age of 50 years compared with selected countries. Data from [18] unless otherwise indicated

Country	Life-time risk at 50 years (%)	
	Women	Men
Sweden	25.6	11.0
South Africa (white)	23.4	7.7
Denmark	23.0	11.3
France	19.3	5.9
China (Hong Kong)	17.7	7.6
USA (Caucasian)	16.1	7.5
Turkey	15.9	3.6
Canada	15.5	5.8
Greece	15.4	6.8
Uzbekistan	14.7	8.7
UK	14.4	5.0
Germany	14.2	5.3
Portugal	13.7	4.8
Finland	12.9	6.0
Kazakhstan	12.6	6.0
Spain	12.6	4.2
Netherlands	12.5	5.4
Singapore (Indian)	12.5	5.2
Bulgaria	11.2	4.4
Qatar ^a^	11.0	8.8
Hungary	10.8	4.2
Poland	10.1	4.2
Moldova	9.3	5.7
Kuwait	9.2	7.6
Abu Dhabi	8.9	8.1
Iran	8.3	5.5
Russia	7.7	3.8
Romania	7.0	3.8
USA (black)	5.9	2.7
Ukraine	5.6	2.9
Saudi Arabia ^b^	4.6	3.7
South Africa (Black)	4.5	1.9
Morocco	4.1	3.1
Botswana	1.1	1.4
Tunisia	0.7	0.7

^a^ Johansson 2021 [19]

^b^ Present study

## Discussion

This study documented the incidence of hip fracture in a representative sample of the population of Saudi Arabia. As expected, hip fractures were more frequent in women than in men. In both sexes, the incidence increased with age. Assuming that the multiregional incidence was similar to the national incidence, Saudi Arabia belongs to the low-risk countries for hip fracture for men and women [21]. There are relatively few studies of fracture rates in Saudi Arabia [22]. In the case of hip fracture, these date from 1991 [23], 2007 [24], and 2013 [25]. All were regional retrospective studies. The most recent was a survey in 24 of 28 hospitals in the Eastern Province. Crude hip fracture rates were approximately 3-fold higher in men and in women than in the present study. Reasons for the disparity are speculative, though the 2013 survey would have captured the large expatriate as well as the indigenous population.

The number of hip fractures nationwide was estimated at 2,949 in 2015. Demographic projections indicate that fracture burden is set to increase markedly in the future. It is estimated that the annual number of hip fractures will increase nearly 7-fold over 35 years. The prediction is relatively robust in that all individuals who will be at the aged of 50 years or more in 2050 are currently adults. However, these estimates may be conservative since they assume that the age- and sex-specific risk of hip fracture remains unchanged over this period. If the age- and sex-specific incidence of hip fracture increases, as has been registered in several countries [26], then the number of fractures may be more than doubled [27]. Such projections are important for healthcare planning.

The incidence of hip fracture is rather similar to that reported for Kuwait and Abu Dhabi. There was, however, a very marked difference in fracture probability between the three countries. The explanation for the difference lies in the assumptions for mortality since fracture probability integrates the fracture hazard with the competing effect of mortality. After the age of 70 years, the mortality estimates were much higher in Saudi nationals than for the nationals of Kuwait or Abu Dhabi. These observations emphasise the importance of the death hazard as well as the fracture hazard in the determination of fracture probability.

Whereas the Saudi FRAX model permits the assessment of fracture probability in Saudi citizens, the question arises of how to assess fracture risk in expatriates. This community comprises 38.3% of the total population, according to the UN data for 2019 and is ethnically very diverse [12]. Current evidence indicates that expatriates retain the risk characteristics of their country of origin [28, 29], so should be assessed as such.

A minority of countries that have a FRAX model also have robust information on the risk of other major osteoporotic fractures. In the absence of such information, FRAX models are based on the assumption that the age- and sex-specific pattern of these fractures is similar to that observed in Malmo [14]. As already noted, this assumption has been shown to be safe in studies reported from many countries [13, 15, 16, 30–32], despite differences in incidence between these countries [21]. This commonality of pattern is supported by register studies, which indicate that in those regions where hip fracture rates are high, so too is the risk of forearm fracture and spine fractures (requiring hospital admission) [33, 34].

The limitations of the present study relate predominately to the accuracy of the FRAX model. This in turn is dependent on the accuracy of the fracture and death hazards used in the construction of the FRAX model. Whereas death rates for the general population are likely to be robust, the hip fractures were garnered from approximately 12% of the population at risk and, despite the care in site selection, may not be representative of the Kingdom. The 12% estimate was based on the number of orthopaedic beds dealing with hip fracture and there is no certainty that all facilities have an equal occupancy of hip fracture cases. It may be relevant, however that hip fracture rates were rather similar to those seen in the neighbouring UAE and Kuwait. It is relevant, however, that, accuracy errors have little impact on the rank order with which the FRAX tool categorizes risk in a given population [35, 36] but they do change the absolute number generated and thus have implications where treatment guidelines are based on cost-effectiveness or the economic burden of disease. In order to address these limitations, population representative of the general population at risk would need to be studied prospectively, preferably over a 10-year time horizon.

In summary, a FRAX model has been created for the Saudi Arabia based on an estimate of the incidence of low-energy hip fractures in a subset of the population. The model should enhance accuracy of determining fracture probability among the Saudi population and help to guide decisions about treatment. Indeed, probability-based assessment of fracture risk using age-specific intervention thresholds has been developed recently for Saudi Arabia to help guide decisions about treatment [37].
